# Circular RNA COL1A2 Mediates High Glucose-Induced Oxidative Stress and Pyroptosis by Regulating MiR-424-5p/SGK1 in Diabetic Nephropathy

**DOI:** 10.1007/s12010-023-04501-1

**Published:** 2023-04-20

**Authors:** Langen Zhuang, Guoxi Jin, Wang qiong, Xiaoxu Ge, Xiaoyan Pei

**Affiliations:** 1 Department of Endocrinology, The First Affiliated Hospital of Bengbu Medical College, No. 287, Changhuai Road, Bengbu, 233004 Anhui China; 2 Department of Endocrinology, Tongren Hospital Affiliated to Jiaotong University, Shanghai, 200000 China

**Keywords:** CircCOL1A2, MiR-424-5p, SGK1, DN

## Abstract

Diabetic nephropathy (DN) represents a major diabetes-related complication, which could undermine renal function. CircCOL1A2 has been previously reported to show abnormal expression during DN. However, its functional role in the progression of DN, as well as the potential molecular mechanisms, remains unclear. The present work examined the expression of circCOL1A2 in the plasma of DN patients, and employed high glucose (HG)-challenged HK-2 cells as the in vitro cell model of hyperglycemia (HG)-induced DN. CircCOL1A2 was silenced using siRNA in HK-2 cells to clarify the functional engagement of circCOL1A2 in HG-induced DN. We examined the roles of circCOL1A2 in regulating oxidative stress by measuring reactive oxygen species (ROS), lipid peroxidation, and superoxide dismutase (SOD) levels. Besides, the effects of circCOL1A2 silencing on pyroptosis were investigated by RT-qPCR, western blot (WB), and ELISA assays. StarBase (version 2.0) was used to identify the downstream effector of circCOL1A2, and their interactions were further verified through dual-luciferase reporter analysis, RNA pull-down assays, and RNA immunoprecipitation (RIP) assay. CircCOL1A2 was highly expressed in DN patients and HG-induced HK-2 cells. Knocking down circCOL1A2 alleviated oxidative stress and pyroptosis upon HG treatment. In addition, we demonstrated that circCOL1A2 knockdown could promote miR-424-5p expression while inhibiting Serum/Glucocorticoid Regulated Kinase 1 (SGK1) level. Furthermore, miR-424-5p inhibitor or SGK1 overexpression impaired the effects of circCOL1A2 knockdown on HG-induced oxidative stress and pyroptosis. Hence, our results demonstrated that the circCOL1A2 mediates HG-exposed pyroptosis and oxidative stress through modulating miR-424-5p/SGK1 axis in diabetic nephropathy, indicating that silencing circCOL1A2 is a potential intervention strategy for DN management.

## Introduction

Diabetes mellitus (DM) represents one cluster of metabolic diseases with the feature of persistently high blood glucose (BG) contents [1]. Type 2 diabetes mellitus (T2DM) is associated with insulin-resistance development and it accounts for 90–95% of DM burdens globally [2]. T2DM-related complications comprise renal failure, heart attack, vision loss, wound repair impairment, and nerve injury [3, 4]. Diabetic nephropathy (DN) represents a major factor for end-stage renal disease (ESRD) worldwide [5, 6]. Typical pathological features in the kidney tissues of DN patients include abnormalities in vascular, tubulointerstitial, and glomerular compartments, which are caused by cell apoptosis and hypertrophy, extracellular matrix (ECM) deposition, and basement membrane thickening. [7]

The uncontrolled high glucose (HG) is the major factor leading to DM-related DN, retinopathy, and cardiomyopathy. Previous studies suggest that oxidative stress can be induced by the excessive production of reactive oxygen species (ROS) in HG-challenged kidney cells [8, 9]. The onset of inflammatory responses upon HG stress could also induce the over-expression of adhesion molecules and chemokines in the proximal tubules to trigger pyroptosis [10–12]. Therefore, HG-induced oxidative stress and inflammatory activation contribute to the occurrence and progression of DN, culminating in the gradual loss of renal function. However, the specific mechanism by which HG triggers oxidative stress and pyroptosis in kidney tissues remains unclear.

Circular RNAs (circRNAs), the non-coding RNAs (ncRNAs) with close-loop structure, are related to pathogenic mechanisms of different disorders [13, 14]. Recently, accumulating evidence pinpoints the critical roles of circRNAs in DN progression [15, 16]. As reporter by Zhang et al.’s study, circ0081108 (circCOL1A2) expression was elevated by 6.85-time in diabetic retinas relative to healthy controls [17]. In addition, circCOL1A2 was also found to become dysregulated in diabetic retinopathy [18]. However, there is currently no studies reporting the roles of circCOL1A2 in HG-induced damages in kidney cells and in the progression of DN occurrence.

MicroRNAs (miRNAs) are important downstream factors mediating the role of circRNAs in pathological conditions. In the studies of DN, circRNA-miRNA interactions have been reported to mediate HG-induced kidney cell damages [19]. For example, circ0000309 is implicated in DN-related ferroptosis regulation by targeting miR-188-3p/GPX4 axis [20]. In addition, circ010383 was reported to act as a molecular sponge for miR-135a to regulate renal fibrosis in DN [21]. Therefore, understanding the role of circRNA-miRNA interaction in HG-induced renal damages is important for develop intervention strategy.

Serum/Glucocorticoid Regulated Kinase 1 (SGK1) is a serine/threonine protein kinase that plays an important role in cellular stress response [22]. This kinase activates certain potassium, sodium, and chloride channels in the regulation of cell survival and renal sodium excretion [23]. High levels of expression of SGK1 is suggested to contribute to pathological conditions such as hypertension and diabetic nephropathy [24, 25]. There is also evidence that SGK1 is engaged in glucose metabolism in the intestine and kidney during the progression of hyperglycemia-induced secondary organ damage [26]. Nerveless, the molecular mechanisms underlying SGK1 dysregulation under hyperglycemia remain to be elucidated.

In the present work, we reported the elevated expression of circCOL1A2 in the plasma samples of DN patients. MiR-424-5p was identified as a miRNA target of circCOL1A2 in regulating HG-induced oxidative stress and pyroptosis in HG-treated renal tubular cells (RTCs). Interestingly, miR-424-5p has been reported to become dysregulated in diabetes-related disorders [27, 28]. Besides, miR-424-5p can regulate the proliferation and extracellular matrix deposition in high glucose-challenged human glomerular mesangial cells (HGMC), suggesting its implication in modulating kidney cell response to hyperglycemia stress [29]. Furthermore, we found that the 3′UTR (untranslated region) of SGK1 mRNA harbors binding sites for miR-424-5p, and miR-424-5p levels could regulate SGK1 expression levels in HG-treated RTCs. Overall, our study highlighted the critical role of circCOL1A2/miR-424-5p/SGK1 axis in modulating HG-induced diabetic nephropathy, suggesting that targeting circCOL1A2 may be a potential intervention strategy for DN management.

## Methods

### Patients and Tissue Samples

Our study protocols gained approval from the Research Ethics Committee of the First Affiliated Hospital of Bengbu Medical College (2021–086). Every participant provided informed consent. A total of 22 DN patients with T2DM and 25 healthy controls from the First Affiliated Hospital of Bengbu Medical College were recruited for this study. Plasma was collected for transcription-quantitative PCR (RT-qPCR) analysis. The diagnosis of diabetes of all patients was confirmed by experienced pathologist. The diagnoses were based on the criteria of the American Diabetes Association (ADA, 2016) [30]. The inclusion criteria of DN patients were as follows: patients age > 18 years; patients who fulfilled the diagnostic criteria of the ADA of 2016; patients with a history of diabetes for more than a year; urinary protein/creatinine ratio > 30 mg/g; persistent urinary protein or renal function decline (GFR < 60 mL/min/1.73m^2^); and biopsy confirmation of diabetes-related renal damages. The inclusion criteria of healthy controls were as follows: no medical history of diabetes; normal fasting blood glucose, renal function, and renal ultrasonography. Exclusion criteria for both groups such as subjects with infectious diseases, chronic systemic inflammatory diseases, blood diseases, autoimmune diseases, malignant tumors, or cardiovascular diseases were excluded.

### Cell Lines and Culture

HK-2 cells lines were obtained in the Cell Bank of Type Culture Collection of Chinese Academy of Sciences (Shanghai, China) and cultivated within DMEM/F12 (HyClone, USA) that contained 10% (vol/vol) fetal bovine serum (FBS, Merck Millipore, Germany) after heat inactivation under the condition of 5% CO_2_ and 37 °C. To mimic hyperglycemia-induced damages in DN, HK-2 cells were cultured for a 2-week period within the medium that contained 30 mM glucose, meanwhile 24.5 mM mannitol combined with 5.5 mM glucose was adopted to be a reference control [31, 32]. Mannitol 24.5 mM was used together with 5.5 mM glucose to maintain the similar osmotic concentration as 30 mM glucose treatment.

### Transfection

Using Lipofectamine 2000 reagent (Invitrogen, Shanghai, China), HK-2 cells were transfected with miR-424-5p inhibitor, miR-424-5p mimic, si-circCOL1A2, SGK1 expression vector, or corresponding controls for a 48-h period, based on the supplier’s instruction. MiRNA mimic and inhibitor were purchased from RiboBio (Guangzhou, China), and si-circCOL1A2, SGK1 expression vector, and corresponding controls were synthesized by Hanheng Biotech (Shanghai, China). Cells were subjected to further analysis 48 h after transfection.

### Measurement of Oxidative Stress Indexes

The measurement of malondialdehyde (MDA) and ROS contents, together with superoxide dismutase (SOD) activity, was conducted with commercially available detection kits following relevant instructions of the supplier. Malondialdehyde (MDA) assay kit (TBA method, A003-1–2), superoxide dismutase (SOD) assay kit (WST-1 method, A001-3–2), and reactive oxygen species (ROS) detection kit (Florescence method, E004) were purchased from Jiancheng Bioengineering Institute (Nanjing, China).

### StarBase

StarBase (version 2.0) was employed for predicting the binding sites between miR-424-5p and circCOL1A2, and the 3′UTR of SGK1 mRNA.

### RT-qPCR

TRIzol reagent (Invitrogen, CA, USA) was adopted for extracting total cellular and plasma RNAs, followed by the cDNA synthesis with ThermoScript RT-PCR synthesis kit (Pittsburgh fermentation company, PA, USA) Thereafter, ABI Prizm step-one plus RT-qPCR System (Applied Biosystems, CA, USA) was applied in qRT-PCR analysis using SYBR Green PCR amplification reagent (Qiagen, WI, USA). The primer sequences used for RT-qPCR were synthesized by Sangon Biotech (Shanghai, China): circCOL1A2, 5′-GACATGCTCAGCTTTGTGGA-3′ (F) and 5′-TGAGAGTCTGCCCTCCAAGT-3′ (R); NLRP3, 5′-GATCTTCGCTGCGATCAACAG-3′ (F) and 5′-CGTGCATTATCTGAACCCCAC-3′ (R); Caspase-1: 5′-TTTCCGCAAGGTTCGATTTTCA-3′ (F) and 5′-GGCATCTGCGCTCTACCATC-3′ (R), IL-1β: 5′-ATGATGGCTTATTACAGTGGGAA-3′ (F) and 5′-GTCGGAGATTCGTAGCTGGA-3′ (R), and GSDMD-N: 5′-GTGTGTCAACCTGTCTATC AAGG-3′ (F) and 5′-CATGGCATCGTAGAAGTGGAAG-3′ (R); and miR-424-5p: 5′-CAGCCACAAA AGAGCACAAT-3′ (F) and 5′-CATGGCATCGTAGAAGTGGAAG-3′ (R); GAPDH was used as an internal reference: 5′-GGTGAAGGTCGGAGTC-3′ (F) and 5′-GAAGATGGTGATGGGATTTC-3′ (R). Relative gene levels were analyzed by 2^−△△Ct^ method.

### Western Blot (WB)

Total protein was extracted from HK-2 cells using RIPA lysis buffer containing protease inhibitor cocktail (Thermo Fisher Scientific, MA, USA). Cells suspended in RIPA buffer were lysed on ice for 10 min and the cell lysate was centrifuged at 12,000 × g for 10 min. Protein concentration was quantified by a BCA Protein Assay Kit (Beyotime Biotechnology, Shanghai, China). Fifteen micrograms of protein was used for SDS-PAGE electrophoresis, and separated proteins were transferred onto the PVDF membrane. After blocking with 5% skimmed milk for 1 h, the membrane was then incubated with primary antibodies (1:1000 dilution, all from Abcam), including NLRP3 (ab263899), Caspase-1 (ab207802), IL-1β (ab254360), GSDMD-N (ab215203), SGK1 (ab32374), and GAPDH (ab8245). After washing with TBS Tween 20, membranes were subject to further probing using HRP-linked secondary antibody (1:3000; Cell signaling technologies, MA, USA). An ECL chemiluminescence kit (Thermo Fisher Scientific, MA, USA) was employed to detect protein bands, and GAPDH was used for internal reference for protein quantification using ImageJ software (Bethesda, MD, USA).

### Enzyme-Linked Immunosorbent Assay (ELISA)

ELISA kits (RD, Minneapolis, MN, USA) were utilized to measure IL-1β and IL-18 concentrations in the cell culture supernatant following specific instructions. Signal development and the optical density of samples and standards were measured at 450 nm using a microplate reader (BioRad, CA, USA). The concentration of each cytokine was measured based on the linear regression of the standards.

### Dual-Luciferase Reporter Assay

The binding sites between miR-424-5p/SGK1 mRNA 3′UTR and miR-424-5p/circCOL1A2 using were predicted using StarBase database. The wild-type (WT) or mutated (MUT) binding sites were cloned into luciferase reporter by Sangon Biotech (Shanghai, China): SGK1 3′UTR WT, SGK1 3′UTR-MUT, circCOL1A2-WT, and circCOL1A2-MUT. The reporter vector was transfected into HK-2 cells for 48 h, together with miRNA mimic or miR-NC. Dual-luciferase reporter assay system (Promega, Madison, WI, USA) was adopted for measuring luciferase activities following specific protocols, with Renilla luciferase being the endogenous reference.

### RNA Pull-Down Assay

This assay was conducted using Pierce™ Magnetic RNA-Protein Pull-Down Kit (#20164, Thermo Fisher Scientific, CA, USA). Biotin-labeled circCOL1A2 (Bio-circCOL1A2 probe) and the control (Bio-NC probe) were synthesized by Sangon Biotech (Shanghai, China). In brief, cell lysates were collected from 1 × 10^6^ HK-2 cells using RIPA lysis buffer, with 10% lysate saving as the input. Thereafter, 200 nM Bio-NC or Bio-circCOL1A2 probe was added to the cell lysate for 1-h incubation, followed by addition of 100 μL streptavidin-magnetic beads. The mixture was incubated for another 2 h, and the associated RNAs on the magnetic beads were purified using TRizol reagent. The relative enrichment of each target was quantified by qRT-PCR.

### RNA Immunoprecipitation (RIP) Assay

This assay was performed by the Magna RIP™ RNA-Binding Protein Immunoprecipitation Kit (#17–700, Millipore, WI, USA) following the supplier’s protocol. First of all, cell lysates were collected from 1 × 10^6^ HK-2 cells using RIPA lysis buffer, with 10% lysate saving as the input. The remaining lysate was subjected to overnight incubation using 100 μL magnetic beads conjugated with 10 μg Immunoglobulin G (IgG control) (ab37355, Abcam) or Argonaute-2 antibody (Ago-2, ab186733, Abcam) under 4℃. After the isolation of the co-precipitated RNA, qRT-PCR was performed to measure circCOL1A2 and miR-424-5p levels.

### Statistical Analysis

Results were displayed as mean ± SD of 3 separate assays. GraphPad Prism 5.0 software was adopted for statistical analyses. We applied unpaired Student’s *t*-test and one-way ANOVA for comparing 2 or multiple groups, respectively. Pearson’s correlation analysis was adopted for determining correlation between the expression levels of two molecules. *P* < 0.05 stood for statistical significance.

## Results

### Increased CircCOL1A2 Level in the Plasma of DN Patients and HG-Induced HK-2 Cells

**Fig. 1:**
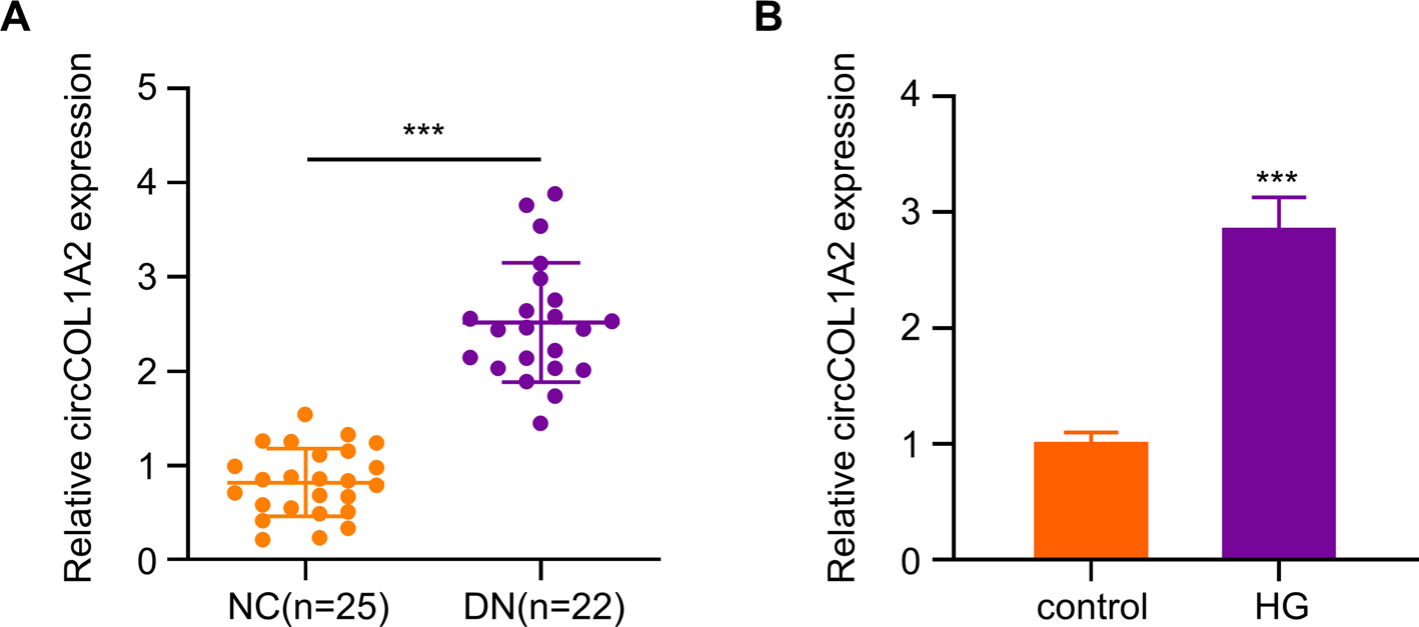
CircCOL1A2 is up-regulated in diabetic nephropathy and HG-induced HK-2 cells. **A** CircCOL1A2 expression was measured through RT-qPCR in healthy controls and DN plasma samples. Three asterisks (***) *P* < 0.001 vs. NC group. **B** CircCOL1A2 expression was measured through RT-qPCR in control and HG-induced HK-2 cells. Three asterisks (***) *P* < 0.001 vs. control

Increased circCOL1A2 expression has been reported in diabetic retinopathy [17, 18]. We wondered whether circCOL1A2 also became dysregulated in DN. We carried out RT-qPCR analysis for detecting circCOL1A2 levels in plasma of healthy people (NC = 25) and diabetic nephropathy patients (DN = 22). The circCOL1A2 expression in DN patients showed an average of 2.5 time increase relative to controls (Fig. 1A). Next, we established an in vitro cell model based on HG-induced HK-2 cells to mimic hyperglycemia condition. HK-2 cells were cultured for a 2-week period within medium containing 30 mM glucose, with the culturing in 5.5 mM glucose plus 24.5 mM mannitol as the control. In HG-challenged HK-2 cells, circCOL1A2 level increased by nearly 3-fold relative to control group (Fig. 1B). These data suggest that elevated circCOL1A2 expression may be implicated in the progression of DN.

### Knocking Down CircCOL1A2 Mitigates Oxidative Stress in HG-Treated HK-2 cells

**Fig. 2:**
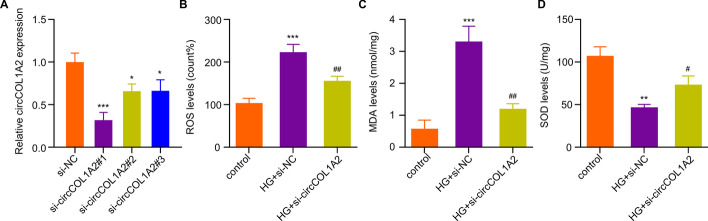
CircCOL1A2 silencing alleviates the oxidative stress in HG-treated HK-2 cells. **A** CircCOL1A2 expression was assessed by RT-qPCR upon the transfection of control siRNA and siRNA targeting circCOL1A2. Three asterisks (***) *P* < 0.001, one asterisk (*) *P* < 0.05 vs. the si-NC group. **B** Level of ROS was measured in control, HG-treated HK-2 cells, and HG-treated HK-2 cells with circCOL1A2 silencing. Three asterisks (***) *P* < 0.001 vs. si-NC group; two number signs (^##^) *P* < 0.01 vs. HG + si-NC group. **C** Level of MDA in control, HG-treated HK-2 cells, and HG-treated HK-2 cells with circCOL1A2 silencing. Three asterisks (***) *P* < 0.001 vs. si-NC group; two number sign (^##^) *P* < 0.01 vs. HG + si-NC group. **D** Level of SOD activity in control, HG-treated HK-2 cells, and HG-treated HK-2 cells with circCOL1A2 silencing. Two asterisks (**) *P* < 0.01 vs. si-NC group; number sign (^#^) *P* < 0.05 vs. HG + si-NC group

To explore the role of circCOL1A2 under hyperglycemia condition, three different circCOL1A2 siRNAs were used to knock down circCOL1A2 in HK-2 cells. RT-qPCR analysis showed that si-circCOL1A2#1 showed the strongest silencing effect, with a 70% reduction of circCOL1A2 when compared to the control siRNA (Fig. 2A). We therefore used this siRNA as si-circCOL1A2 in subsequent studies. Next, we measured ROS level as the indicator of oxidative stress level in HK-2 cells. The results showed that HG treatment increased ROS production by more than 2-fold in HK-2 cells, while silencing circCOL1A2 significantly reduced ROS production (Fig. 2B). Likewise, the analysis of the lipid peroxidation by-product MDA revealed a 3-fold increase of MDA production upon HG challenge, and silencing circCOL1A2 suppressed HG-induced MDA level by more than 60% (Fig. 2C). In contrast, the ROS detoxification enzyme SOD activity was inhibited by 50% upon HG treatment, in si-circCOL1A2 partially restored SOD activity upon HG treatment (Fig. 2D). Taken together, silencing circCOL1A2 could alleviate HG-induced oxidative stress in HK-2 cells.

### Knocking Down CircCOL1A2 Inhibits Pyroptosis in HG-Treated HK-2 Cells

**Fig. 3:**
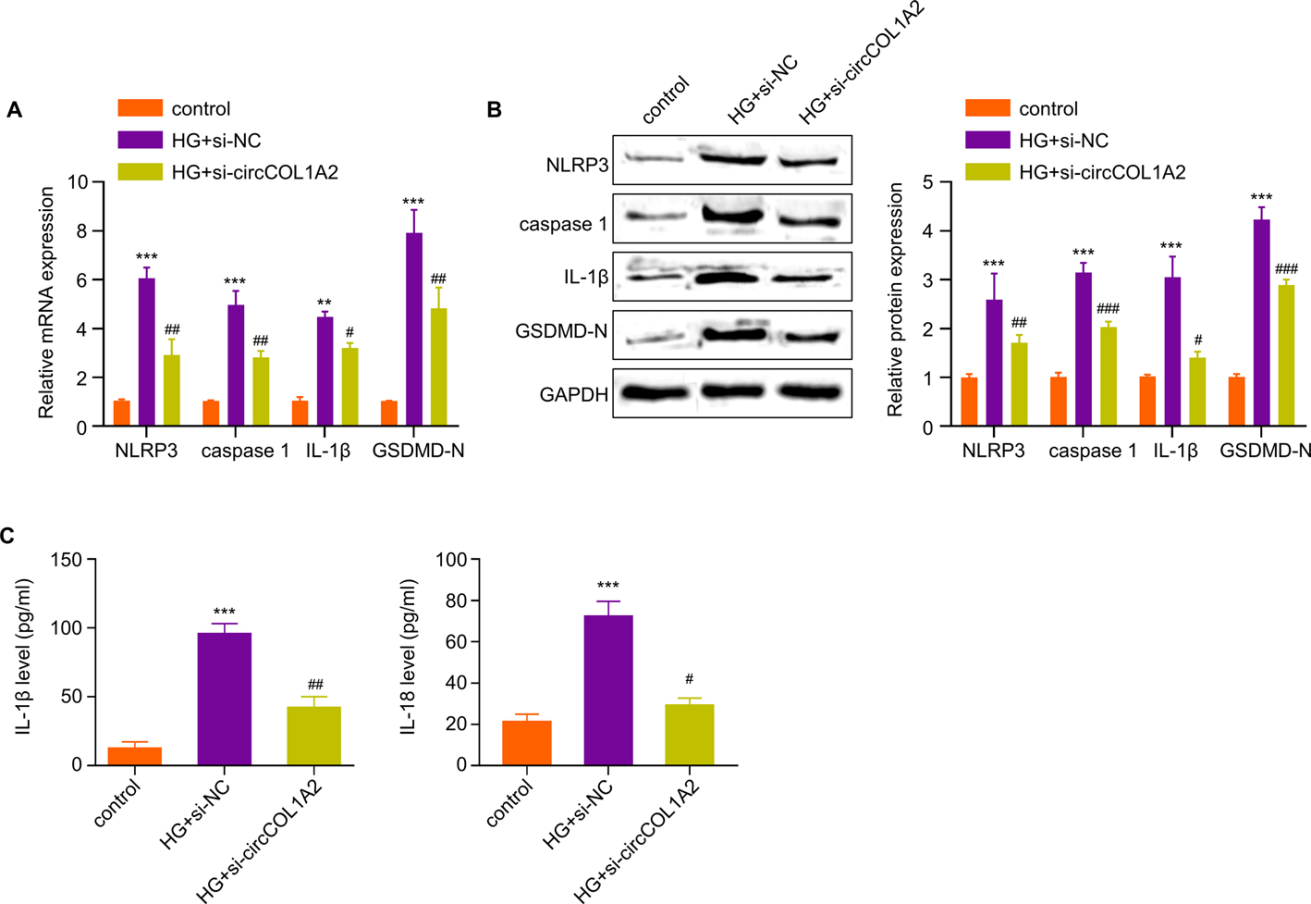
CircCOL1A2 silencing suppresses HG-induced HK-2 cell pyroptosis. **A** RT-qPCR was carried out for measuring Caspase-1, NLRP3, IL-1β and GSDMD-N mRNA levels in control, HG-treated HK-2 cells, and HG-treated HK-2 cells with circCOL1A2 silencing. Two number signs (^##^) *P* < 0.01, one number sign (^#^) *P* < 0.05 vs. HG + si-NC group; three asterisks (***) *P* < 0.001, two asterisks (**) *P* < 0.01 vs. control. **B** Caspase-1, NLRP3, GSDMD-N and IL-1β protein levels were assessed through WB assay in control, HG-treated HK-2 cells, and HG-treated HK-2 cells with circCOL1A2 silencing. Three number signs (^###^) *P* < 0.001, two number signs (^##^) *P* < 0.01, one number sign (^#^) *P* < 0.05 vs. HG + si-NC group; three asterisks (***) *P* < 0.001 vs. control. **C** IL-18 and IL-1β expression was assessed through ELISA in control, HG-treated HK-2 cells, and HG-treated HK-2 cells with circCOL1A2 silencing. Two number signs (^##^) *P* < 0.01, one number sign (^#^) *P* < 0.05 vs. HG + si-NC group; three asterisks (***) *P* < 0.001 vs. control

Subsequently, the present work examined the effect of silencing circCOL1A2 on HG-mediated pyroptosis. Pyroptosis-associated proteins, including Caspase-1, NLRP3, GSDMD-N and IL-1β, and their corresponding mRNA levels were examined by WB and RT-qPCR upon HG treatment with or without circCOL1A2 silencing. At the mRNA level, HG treatment led to the upregulation of Caspase-1 (6-fold), NLRP3 (5-fold), GSDMD-N (4-fold), and IL-1β (8-fold) in HK-2 cells; however, silencing circCOL1A2 significantly suppressed their upregulation upon HG treatment (Fig. 3A). At the protein level, HG treatment led to the upregulation of Caspase-1 (2.5-fold), NLRP3 (3-fold), GSDMD-N (3-fold), and IL-1β (4.5-fold) in HK-2 cells, and silencing circCOL1A2 significantly reduced their protein levels upon HG treatment (Fig. 3B). Similarly, ELISA measurement in the cell culture supernatant revealed a 10-fold increase of IL-1β and a 3.5-fold increase of IL-18 production upon HG treatment, but their production was inhibited by circCOL1A2 silencing (Fig. 3C). These results suggest silencing circCOL1A2 shows a protective effect against HG-induced pyroptosis.

### CircCOL1A2 Modulates MiR-424-5p Expression in HK-2 Cells

**Fig. 4:**
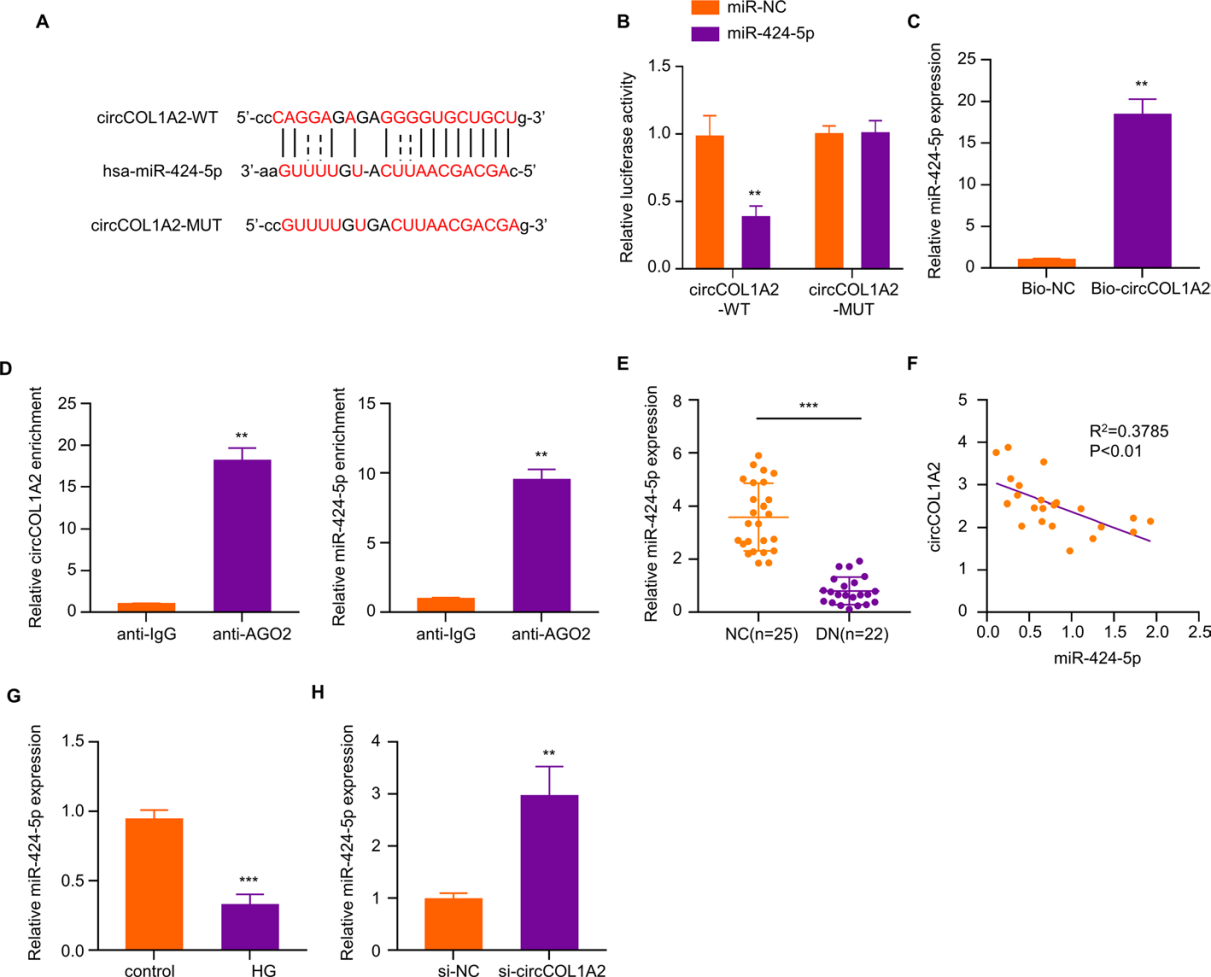
CircCOL1A2 silencing promotes miR-424-5p expression. **A** StarBase was adopted for predicting binding sites between miR-424-5p for circCOL1A2. **B** Dual-luciferase reporter assay was conducted for detecting luciferase activities of circCOL1A2-WT or circCOL1A2-MUT reporter in the presence of miR-424-5p mimic or miR-NC co-transfection. Two asterisks (**) *P* < 0.01 vs. miR-NC group. **C** Bio-NC or Bio-circCOL1A2 probe was utilized to precipitate miR-424-5p in RNA pull-down analysis, followed by RT-qPCR quantification. Two asterisks (**) *P* < 0.01 vs. Bio-NC group. **D** Association of circCOL1A2 and miR-424-5p in Ago-2 protein complex was verified through RIP assay. Two asterisks (**) *P* < 0.01 vs. anti-IgG group. **E** miR-424-5p expression was measured through RT-qPCR in the plasma samples of control and DN patients. Three asterisks (***) *P* < 0.001 vs. NC group. **F** Spearman correlation coefficient analysis of circCOL1A2 and miR424-5p in DN patient samples. **G** miR-424-5p expression was measured through RT-qPCR in control and HG-treated HK-2 cells. Three asterisks (***) *P* < 0.001 vs. control group. **H** miR-424-5p expression was determined through RT-qPCR in HK-2 cells transfected with control or cicCOL1A2 siRNA. Three asterisks (***) *P* < 0.001 vs. si-NC group

We next sought to search for the miRNA target of circCOL1A2, since circRNAs frequently regulate downstream miRNA to regulate cellular processes^.^ [13, 14] it is unknown the mechanism of the circCOL1A2 effect in diabetic nephropathy. StarBase (version 2.0) analysis showed that there exist complementary binding sites between circCOL1A2 and miR-424-5p (Fig. 4A). We next used luciferase reporter containing circCOL1A2 WT or MUT binding sites in the dual-luciferase reporter assay. The co-transfection of miR-424-5p mimic reduced the luciferase activity of the WT reporter by more than 50%, while there was no inhibitory effect on the MUT reporter, indicating the interaction between circCOL1A2 and miR-424-5p at predicted bindings sites (Fig. 4B). RNA pull-down assay revealed that miR-495-3p could be precipitated with Bio-circCOL1A2 probe by more than 15-fold relative to the Bio-NC probe (Fig. 4C). In the meantime, according to RIP assay, miR-495-3p and circCOL1A2 could be co-precipitated by Ago-2 antibody compared to the IgG control (Fig. 4D). These results suggest the functional and physical interaction between circCOL1A2 and miR-424-5p.

Furthermore, this work analyzed miR-424-5p levels in plasma of healthy people (NC = 25) and diabetic nephropathy patients (DN = 22). In contrast to the increased circCOL1A2 expression, the average miR-424-5p level was reduced by more than 3-fold in DN patients in comparison to the healthy controls (Fig. 4E). Meanwhile, there was a significant negative correlation between circCOL1A2 level and miR-424-5p expression within plasma samples of DN patients (Fig. 4F). In HG-treated cells, miR-424-5p was reduced by more than 50% relative to the control (Fig. 4G), and circCOL1A2 knockdown caused a 3-fold increase of miR-424-5p in HK-2 cells (Fig. 4H). These results further indicated that circCOL1A2 negatively regulates miR-424-5p expression.

### MiR-424-5p Targets SGK1 in HK-2 Cells

**Fig. 5:**
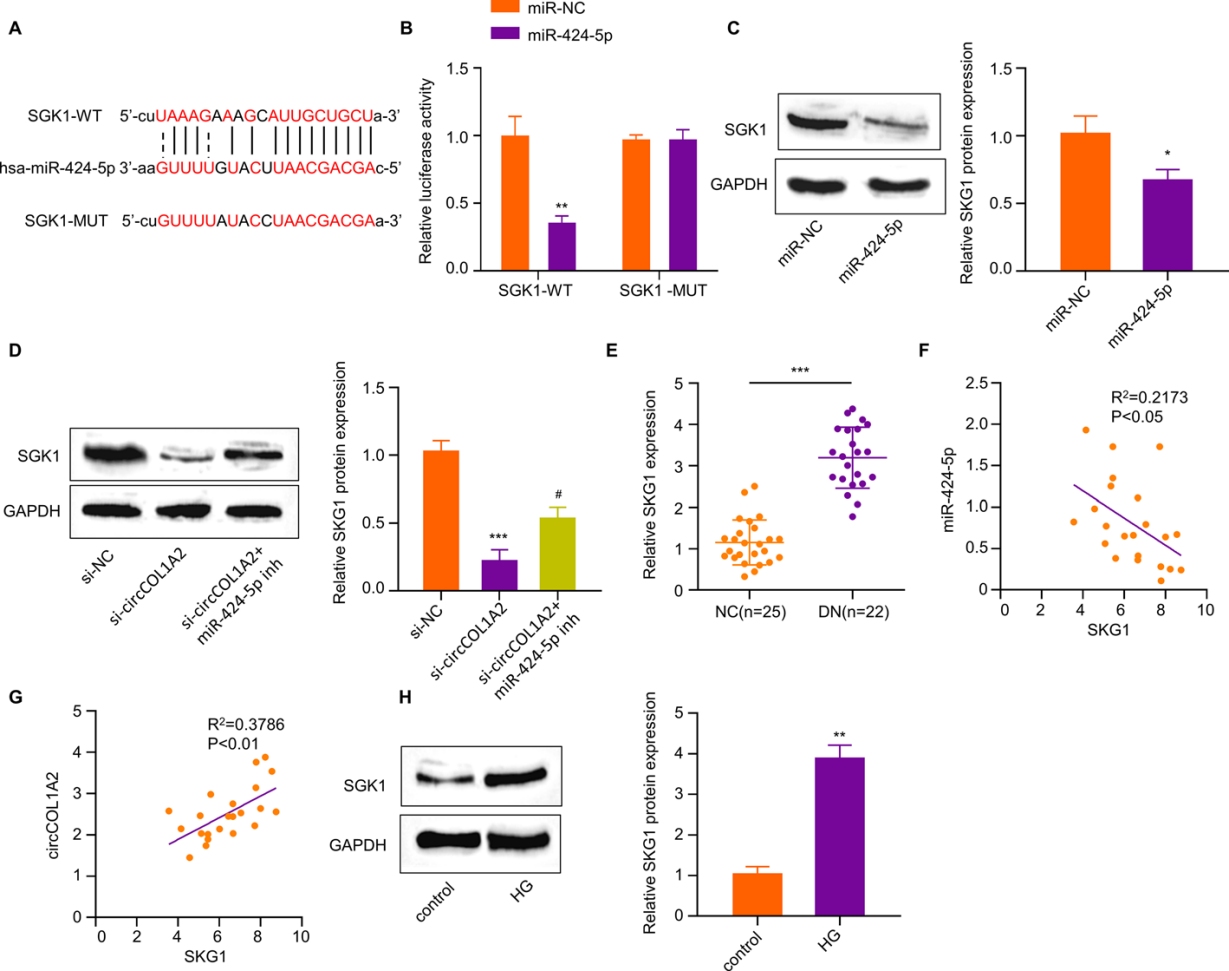
MiR-424-5p targets SGK1 in HK-2 cells. **A** StarBase was utilized for predicting bindings sites between SKG1 mRNA 3’ UTR and miR-424-5p. **B** Dual-luciferase reporter assay was carried out using SGK1-WT/SGK1-MUT reporter in the presence of miR-424-5p mimic or miR-NC co-transfection. Two asterisks (**) *P* < 0.01 vs. miR-NC group. **C** SGK1 protein level was evaluated through WB assay in HK-2 cells transfected with miR-NC or miR-424-5p mimic. One asterisk (*) *P* < 0.05 *vs.* miR-NC group. **D** SGK1 protein level was evaluated through WB assay in HK-2 cells transfected with circCOL1A2 siRNA with or without miR-424-5p inhibitor. Number sign (^#^) *P* < 0.05 vs. si-circCOL1A2 group; three asterisks (***) *P* < 0.001 *vs*. miR-NC group. **E** SGK1 level was evaluated through RT-qPCR in DN patient and healthy control plasma samples. Three asterisks (***) *P* < 0.001 vs. NC. **F** Spearman correlation coefficient analysis of miR-424-5p and SGK1 mRNA level. **G** Spearman correlation coefficient analysis of circCOL1A2 level and SGK1 mRNA level. **H** SGK1 protein level was assessed through WB assay in control and HG-treated cells. Two asterisks (**) *P* < 0.01 *vs*. control

To further explore the downstream mechanism of miR-424-5p, this work employed StarBase (version 2.0) to predict mRNA target of miR-424-5p. According to our results, the 3′UTR (untranslated region) of SGK1 mRNA harbors binding sites for miR-424-5p (Fig. 5A), suggesting that SGK1 mRNA was a potential target of miR-424-5p. Moreover, dual-luciferase reporter assay verified the interaction of miR-424-5p with SGK1 mRNA, since SGK1-WT reporter could be suppressed by miR-424-5p mimic, but SGK1-MUT reporter did not exhibit any significant change by miR-424-5p mimic (Fig. 5B). MiR-424-5p over-expression by its mimic in HK-2 cells decreased the SGK1 protein level by 40% (Fig. 5C). Furthermore, we also showed that circCOL1A2 silencing dramatically repressed SGK1 expression, while the co-administration of miR-424-5p inhibitor partially restored SGK1 level (Fig. 5D).

Next, we explored the expression level of SGK1 in the plasma samples of healthy people (NC = 25) and diabetic nephropathy patients (DN = 22). SGK1 mRNA level was increased by nearly 3-fold in DN patients (Fig. 5E). According to correlation analysis, miR-424-5p level was adversely associated with SGK1 mRNA level in plasma samples of DN patients (Fig. 5F). In contrast, there was a significant positive correlation between circCOL1A2 expression and SGK1 mRNA level (Fig. 5G). We further showed that SGK1 protein level was increased by nearly 4-fold within HG-treated HK-2 cells (Fig. 5H). Therefore, circCOL1A2/miR-424-5p axis regulates SGK1 expression in HK-2 cells.

### CircCOL1A2 Regulates HG-Induced Damages in HK-2 Cells Through MiR-424-5p/SGK1 Axis

**Fig. 6:**
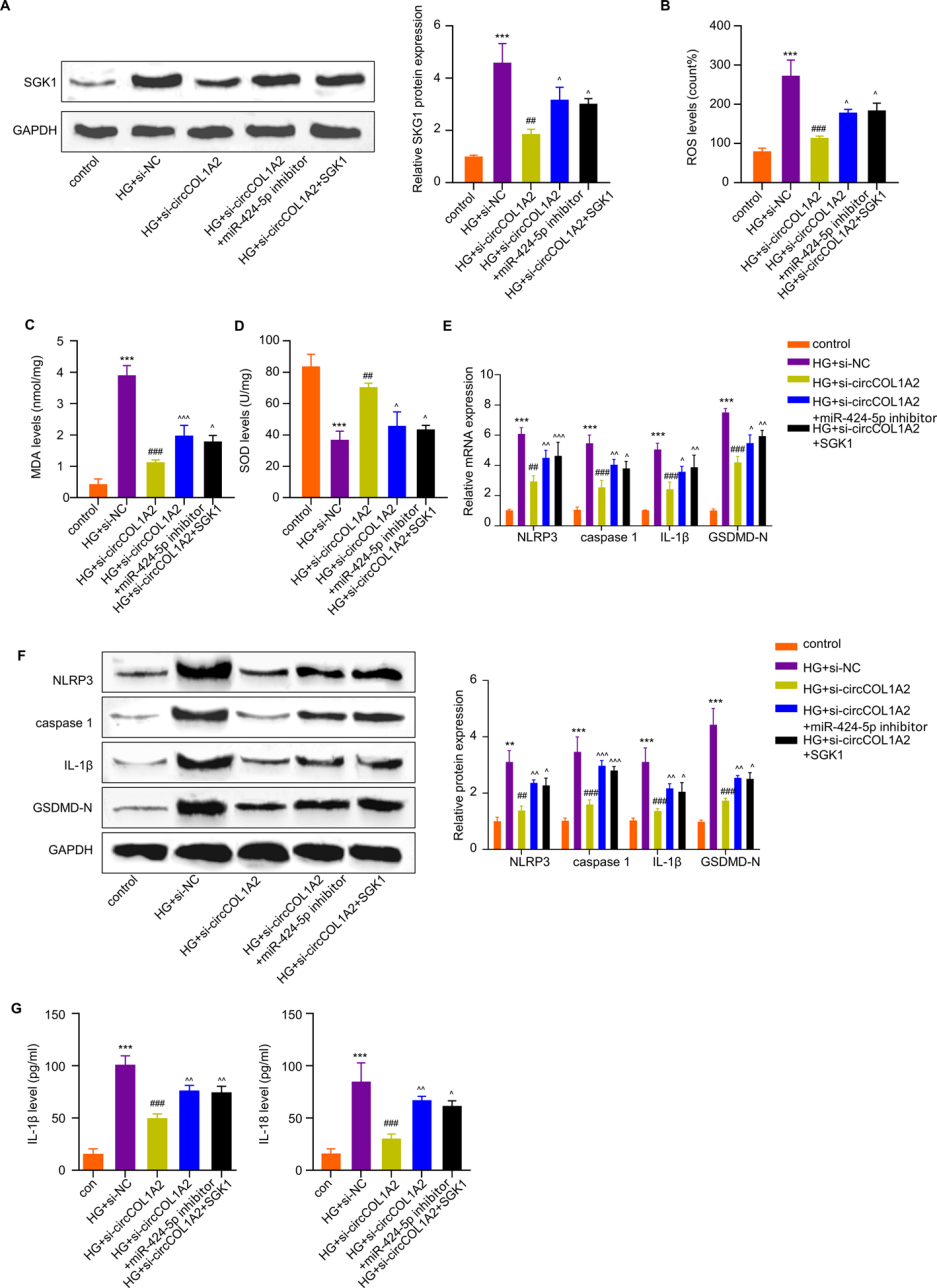
CircCOL1A2 regulates HG-triggered HK-2 cells injury through miR-424-5p/SGK1 axis. The following experimental groups were established in HK-2 cells: control, HG treatment, HG + circCOL1A2 silencing, HG + circCOL1A2 silencing plus miR-424-5p inhibitor, and HG + circCOL1A2 silencing plus SGK1 expression vector. **A** SGK1 protein level was assessed through WB assay. Circumflex accent (^) *P* < 0.05 vs. HG + circCOL1A2 group; two number signs (^##^) *P* < 0.01 vs. HG + si-NC group; three asterisks (***) *P* < 0.001 vs. control. **B**–**D** Level of ROS, MDA and SOD activities were measured using corresponding commercial kits. Three asterisks (***) *P* < 0.001 vs. the control group; three number signs (^###^) *P* < 0.001, two number signs (^##^) *P* < 0.01 vs. the HG + si-NC group; three circumflex accent (^^^) *P* < 0.001, one circumflex accent (^) *P* < 0.05 vs. the HG + circCOL1A2 group. **E** NLRP3, Caspase-1, IL-1β, and GSDMD-N expression levels were assessed by RT-qPCR. Three asterisks (***) *P* < 0.001 vs. the control group; three number signs (^###^) *P* < 0.001, two number signs (^##^) *P* < 0.01 vs. the HG + si-NC group; three circumflex accent (^^^) *P* < 0.001, two circumflex accent (^^) *P* < 0.01, one circumflex accent (^) *P* < 0.05 vs. the HG + circCOL1A2 group. **F** The protein expression levels of NLRP3, Caspase-1, IL-1β, and GSDMD-N were evaluated by Western blot. Three asterisks (***) *P* < 0.001, two asterisks (**) *P* < 0.01 vs. the control group; three number signs (^###^) *P* < 0.001, two number signs (^##^) *P* < 0.01 vs. the HG + si-NC group; three circumflex accent (^^^) *P* < 0.001, two circumflex accent (^^) *P* < 0.01, one circumflex accent (^) *P* < 0.05 vs. the HG + circCOL1A2 group. **G** IL-18 and IL-1β expression was assessed through ELISA. Three asterisks (***) *P* < 0.001 vs. the control group; three number signs (^###^) *P* < 0.001 vs. the HG + si-NC group; two circumflex accent (^^) *P* < 0.01, one circumflex accent (^) *P* < 0.05 *vs*. the HG + circCOL1A2 group

To validate whether miR-424-5p/SGK1 axis mediates the effect of circCOL1A2 on HG-induced cell injury in HK-2 cells, we established the following experimental groups: control, HG treatment, HG + circCOL1A2 silencing, HG + circCOL1A2 silencing plus miR-424-5p inhibitor, and HG + circCOL1A2 silencing plus SGK1 expression vector. WB analysis showed that HG treatment caused a dramatic over-expression of SGK1, and silencing circCOL1A2 significantly suppressed the over-expression of SGK1. Meanwhile, the co-administration of miR-424-5p inhibitor or SGK1 expression vector impaired the effect of circCOL1A2 silencing (Fig. 6A).

ROS measurement showed that the protective effect of circCOL1A2 knockdown on HG-induced oxidative stress was impaired by SGK1 expression or miR-424-5p inhibitor (Fig. 6B). Similar results were observed with lipid peroxidation by-product MDA measurement (Fig. 6C). Consistently, silencing circCOL1A2 could increase SOD activity upon HG challenge, while SGK1 expression vector or miR-424-5p inhibitor suppressed this effect (Fig. 6D). These results suggest that circCOL1A2 impinges on the redox homeostasis of HG-induced HK-2 cells via mir-424-5p/SGK1 pathway.

Furthermore, we performed WB and qRT-PCR assays to detect pyroptosis-associated factors in above experiment groups. As shown in Fig. 6 E and F, si-circCOL1A2 transfection suppressed the upregulation of pyroptosis markers in HG-treated HK-2 cells, and miR-424-5p inhibitor or SGK1 over-repression weakened this effect. Similarly, we found that the inhibitory effect of circCOL1A2 silencing on HG-triggered IL-18 and IL-1β production was dramatically impaired following SGK1 expression and miR-424-5p inhibition (Fig. 6G). Collectively, circCOL1A2 modulates HG-induced HK-2 cell injury via miR-424-5p/SGK1 pathway.

## Discussion

DN has become a prevalent disorder in diabetic patients and has a complicated pathogenic mechanism during its progression into ESRD [33]. Accompanied by high morbidity and mortality rates, DN has imposed tremendous physico-mental pressure and financial burdens on the patients and the society. The uncontrolled hyperglycemia has been recognized as a major risk factor for diabetic-induced nephropathy. As previously reported, chronic hyperglycemia could exacerbate ROS production and oxidative stress in kidney [8, 9] and further induces pyroptosis [10, 11]. Nonetheless, the precise mechanisms by which HG triggers oxidative and inflammatory damages in renal tissues is still largely unclear.

The present work examined the mechanism underlying HG-triggered DN using HG-exposed HK-2 cells and DN patient plasma samples. Recent works have highlighted the crucial roles of circRNAs in regulating the progression of DN [15, 16]. Through literature review, a previous study reported that circCOL1A2 expression level increased by 6.85-fold in patients with diabetic retinopathy relative to non-diabetic counterparts [17]. In addition, circCOL1A2 could promote angiogenesis via regulating miR-29b/VEGF axis in diabetic retinopathy [18]. Our data provided novel evidence regarding the role of circCOL1A2 in HG-induced damages in renal tubular cells. CircCOL1A2 expression is markedly elevated in HG-treated HK-2 cells and the plasma samples of DN patients. Subsequently, this work showed that silencing circCOL1A2 level via siRNA in HK-2 cells could mitigate HG-induced oxidative stress and pyroptosis. The knockdown of circCOL1A2 reduced ROS production within HG-treated HK-2 cells and pyroptosis-related protein markers such as Caspase-1, GSDMD-N, NLRP3, and IL-1β were heavily upregulated upon HG challenge. ELISA measurement of IL-1β and IL-18 in the supernatant from HK-2 cells showed a protective effect of circCOL1A2 silencing on inflammatory response. Therefore, targeting circCOL1A2 may be employed as an intervention strategy to mitigate HG-induced damages in DN.

StarBase (version 2.0) was used to identify miR-454-3P as a target of circCOL1A2. Their interaction was verified by RIP, dual-luciferase reporter, and RNA pull-down assays. Previous studies have revealed versatile roles of miR-424-5p on dictating cellular sensitivity towards cell ferroptosis, apoptosis, and oxidative stress [34–36]. Interestingly, miR-424-5p level was found to be decreased during DN [29]. In agreement with this, we also showed the reduced miR-424-5p in DN patients and HG-induced HK-2 cells. Furthermore, knocking down circCOL1A2 could promote miR-424-5p level, and there was a negative correlation between circCOL1A2 and miR-424-5p levels within plasma from patients with DN. Moreover, the application of miR-424-5p inhibitor was able to undermine the protective effect of circCOL1A2 silencing on HG-induced HK2 cell damages. Thus, miR-424-5p at least partially mediates the protective effect of circCOL1A2 on HG-induced HK2 cell damages.

SGK1 mRNA was further confirmed as a target of miR-424-5p. Our study reported that SGK1 mRNA level in DN patient samples was significantly increased, and miR-424-5p over-expression reduced the SGK1 expression. As revealed by correlation analysis, miR-424-5p level was adversely associated with SGK1 level within plasma of DN patients, while circCOL1A2 level seems to be positively correlated with SGK1 expression. Through experimental analysis, we further demonstrated that circCOL1A2 regulates the HG-induced HK-2 cell injury through miR-424-5p/SGK1 axis. Previous studies have found that SGK1 could mediate cell autophagy, apoptosis, and oxidative stress [37–39]. A previous study reported the increased level of SGK1 in DN patients, and inhibiting SGK1 promotes autophagy in renal tubular epithelial cells [40]. High levels of expression of SGK1 is also considered a contributing factor to pathological conditions such as hypertension and diabetic nephropathy [24, 25]. There is also evidence that SGK1 is implicated in glucose metabolism in the kidney during the progression of hyperglycemia-induced organ damage [26]. Thus, the over-expression of SGK1 in DN patients regulated by elevated circCOL1A2 level may contribute to renal tissue damages due to chronic hyperglycemia.

However, the protective effect of silencing circCOL1A2 in mitigating hyperglycemia-induced renal damages needs further validation in the animal model of DN. Besides, the mechanism by which hyperglycemia induces circCOL1A2 upregulation needs to be elucidated. Equally important, how SGK1 regulates ROS production and pyroptosis under hyperglycemia challenge warrants further investigations.

## Conclusion

In conclusion, we reported the over-expression of circCOL1A2 in DN patients and HG-challenged HK-2 cells. Knocking down circCOL1A2 suppressed HG-induced pyroptosis and oxidative stress in HK-2 cells. Mechanistically, circCOL1A2 knockdown restrains HG-induced oxidative stress and pyroptosis by promoting miR-424-5p expression, thereby inhibiting SGK1 expression. Our study provides novel insights into the mechanisms underlying SGK1 upregulation in DN progression, and highlights circCOL1A2 as a potential target for therapeutic intervention in DN management.

## Data Availability

The data is available upon reasonable request.

## Abbreviations

T2DM: Type 2 diabetes mellitus
DN: Diabetic nephropathy
ESRD: End-stage renal disease
HG: High glucose
ROS: Reactive oxygen species;
circRNAs: Circular RNAs
RT-qPCR: Reverse transcription-quantitative
MDA: Malondialdehyde
SOD: Superoxide dismutase
RIP: RNA immunoprecipitation
SDS–PAGE: Sodium dodecyl sulfate polyacrylamide gel electrophoresis
PVDF: Polyvinylidene difluoride
SD: Standard deviation
